# Comparison between covert sound-production task (sound-imagery) vs. motor-imagery for onset detection in real-life online self-paced BCIs

**DOI:** 10.1186/s12984-020-0651-4

**Published:** 2020-02-07

**Authors:** Youngjae Song, Francisco Sepulveda

**Affiliations:** BCI-Neural Engineering Group – School of Computer Science and Electronic Engineering, University of Essex, Colchester, UK

**Keywords:** Brain-computer interface, Onset detection, Self-paced BCI, Sound-production imagery

## Abstract

**Background:**

Even though the BCI field has quickly grown in the last few years, it is still mainly investigated as a research area. Increased practicality and usability are required to move BCIs to the real-world. Self-paced (SP) systems would reduce the problem but there is still the big challenge of what is known as the ‘onset detection problem’.

**Methods:**

Our previous studies showed how a new sound-imagery (SI) task, *high-tone covert sound production*, is very effective for onset detection scenarios and we expect there are several advantages over most common asynchronous approaches used thus far, i.e., motor-imagery (MI): 1) Intuitiveness; 2) benefits to people with motor disabilities and, especially, those with lesions on cortical motor areas; and 3) no significant overlap with other common, spontaneous cognitive states, making it easier to use in daily-life situations. The approach was compared with MI tasks in online real-life scenarios, i.e., during activities such as watching videos and reading text. In our scenario, when a new message prompt from a messenger program appeared on the screen, participants watching a video (or reading text, browsing images) were asked to open the message by executing the SI or MI tasks, respectively, for each experimental condition.

**Results:**

The results showed the SI task performed statistically significantly better than the MI approach: 84.04% (SI) vs 66.79 (MI) True-False positive rate for the sliding image scenario, 80.84% vs 61.07% for watching video. The classification performance difference between SI and MI was found not to be significant in the text-reading scenario. Furthermore, the onset response speed showed SI (4.08 s) being significantly faster than MI (5.46 s). In terms of basic usability, 75% of subjects found SI easier to use.

**Conclusions:**

Our novel SI task outperforms typical MI for SP onset detection BCIs, therefore it would be more easily used in daily-life situations. This could be a significant step forward for the BCI field which has so far been mainly restricted to research-oriented indoor laboratory settings.

## Introduction

Even though the BCI field has quickly developed in the last few years, it is mainly investigated as a research area due to shortcomings in terms of practicality and usability. Many BCI systems employ cue-based (synchronous) approaches, where the analysis and classification of brain signals are locked to the machine’s predefined timing protocol [1]. This means that it forces users to follow the computer’s timing commands (locked to the machine). Event-related approaches such as P300 and SSVEP also require users to keep their mental focus and/or gaze on the computer interface for long periods of time, which is not only unnatural, but also leads to loss of both user autonomy and the ability to have a rich interaction with their environment [1–4]. These, along with still prevailing reliability limitations, are the main issues when BCIs are used outside laboratory settings.

On the other hand, self-paced (asynchronous) systems enable users to control the system in a more natural way, i.e., according to their own timing and speed of communication without any computer-controlled stimulus [5]. Giving users more autonomy and flexibility in terms of system control is integral to the ultimate aim of utilizing BCIs in the real world [4].

However, self-paced approaches usually requires more complex analyses and have worse correct classification rates as well as a more complex system design compared to cue-based systems due to the lack of knowledge about precise time location of the user commands. That is, the problem of *when* a relevant spontaneous event happens supersedes the problem of *what* command was given by the user. The system must thus first identify a specific active task against the idle (i.e., no specific control) state [6]. However, determining the time of spontaneous command onset, the so-called ‘onset detection problem’, is difficult as there is no direct non-invasive means to validate the timing of the onset event. Thus, onset detection systems have an inherent timing error, which has recently been reduced to a promising few seconds [2, 4]. Nonetheless, onset detection can be used as an on/off switch for self-paced BCIs [7].

In this paper, a novel sound-production related cognitive task (sound-production imagery, SI), i.e., high tone covert sound production, which showed promising onset detection results in our previous offline setting studies [2, 4, 6, 8], was compared with the still most common approach for online self-paced onset detection systems in real-life scenarios, i.e., motor imagery (MI). As the self-paced covert sound-production task is a new concept that we recently proposed, there is no literature (other than our own) related to onset detection in BCIs used with covert speech or sound-production related tasks. In [9–11] the authors investigated a somewhat similar imaginary speech case, but it was done in a cue-based system, i.e., not for onset detection. In that study the subjects were asked to think either the syllable ‘ba’ or ‘ku’ at a specific rhythm with audio cues. In addition, other speech related EEG-based BCI studies using different syllables (or vowels), e.g., [12], are focused on the discrimination between various tasks and not on onset detection (i.e., idle versus intentional state).

In the recent self-paced system, MI is mostly used (e.g., [1, 3, 13–17]). However, MI self-paced onset detection systems have a crucial issue when they are used outside laboratory settings. The mental procedure is largely overlapping with other common, spontaneous cognitive states. For example, a classifier would not be able to reliably identify whether the onset detection was from an actual relevant command or from other daily-life gestures such as waving, head movements, etc., especially if MI is also used for multi-class control (i.e., not for onset detection) within the same BCI system. This has motivated the search for alternative cognitive tasks for self-paced BCIs. Thus, the MI vs SI comparison in daily-life conditions will be discussed in this paper.

A Sound-production related cognitive task is also needed to reduce the chances of intentional command (IC) false positives but this can be addressed by choosing cognitive tasks that do not significantly overlap with other common, spontaneous and frequent cognitive states [6]. Using specific words, syllables or letters for the onset detection would likely increase both the onset false positives as well as the task-related false negatives due to the large overlap with the continuous internal speech in normal thought processes. For this reason, we have chosen high tone sound production as an onset switch as this task is unlikely to overlap with normal thought processes. We also expected the chosen task to be easy to produce and control voluntarily and there is no dependence on the users’ mother-language or even on their language capabilities. In addition, the SI task used here is expected to be very intuitive for the vast majority of people as we almost constantly ‘speak’ internally while awake. This is also a big advantage for people with severe motor disabilities, especially those with damage in motor control cortical areas, an important target population for BCIs [4]. Besides these advantages, our approach showed significantly better performance results than the MI task for self-paced onset detection BCI, which will be discussed in more detail below. Bringing BCI to the real world and maintaining user autonomy and engagement with the surroundings as much as possible is important and this is the main novelty of the present paper.

## Methodology

### Cognitive tasks description

In this experiment, two different cognitive tasks were tested for the sake of comparison. One was MI, which is a typically used mental task in the BCI field. It is performed by the imagining of limb movements such as those of the hands, feet or the tongue [5]. In our experiment, participants were instructed to imagine the movement of their primary wrist.

The other task was a sound-production related cognitive task (Sound Imagery (SI) proposed in our previous studies [2, 4, 6, 8]). The task had showed encouraging results in an offline semi self-paced onset detection system. For this reason, we used this SI task in an online experiment in order to test it in real-life task scenarios and to compare it with a typically used MI task. In this experiment, participants were instructed to imagine producing an ‘um’ sound with a high tone in a covert (i.e. imaginary) manner, which necessarily overlaps with auditory recall (auditory imagery [18]). In addition, participants were told not to tense any organs related to the sound-production in order to ensure the purely covert task execution. The high pitch tone level was chosen by the participants based on sounds they could comfortably generate for a couple of seconds but high enough to think that they were unusual tones to be used in a normal daily life situation.

As this experiment was about online self-paced onset detection, the idle state (i.e., non-control or null state) had to be defined for training purposes so that the Intentional-Control (IC) task state could be reliably distinguished from the idle state. To this end, participants were instructed not to think of any IC tasks and to stay calm and relaxed for the idle state recordings.

### Experimental paradigm

Twelve healthy subjects (Ten males and two females aged between 19 and 27) with normal or corrected vision participated in the experiments. Four of them (P3, P4, P10 and P12) had previous experience in other BCI experiments and the remaining eight were naïve subjects. Each subject sat comfortably on a medical chair and a monitor was placed 50 cm away from the subject. A keyboard was placed on their lap so that they could give feedback to the system. The experiments were conducted in accordance with the University of Essex Ethics Committee guidelines.

**Fig. 1:**
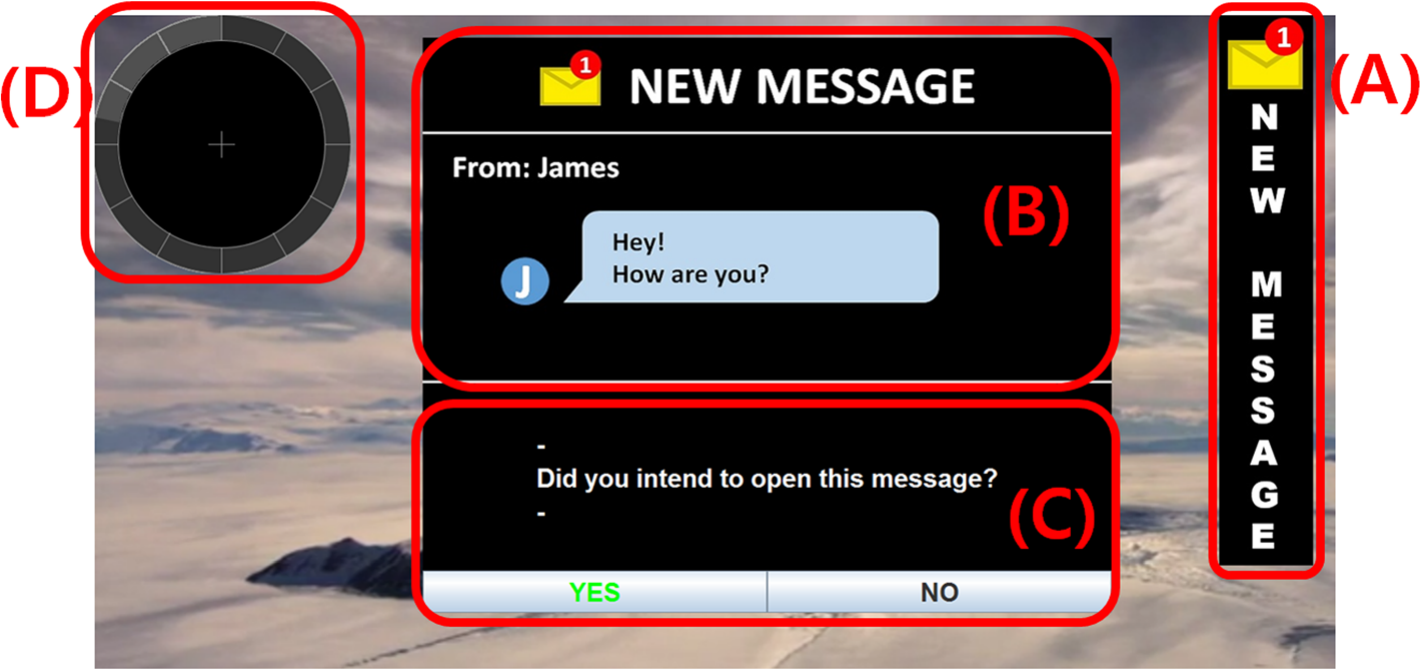
Messaging system interface example from the experiment. (**a**): new message alert, (**b**): message dialogue, (**c**): user feedback panel and (**d**): time keeping interface) [8]

**Fig. 2:**
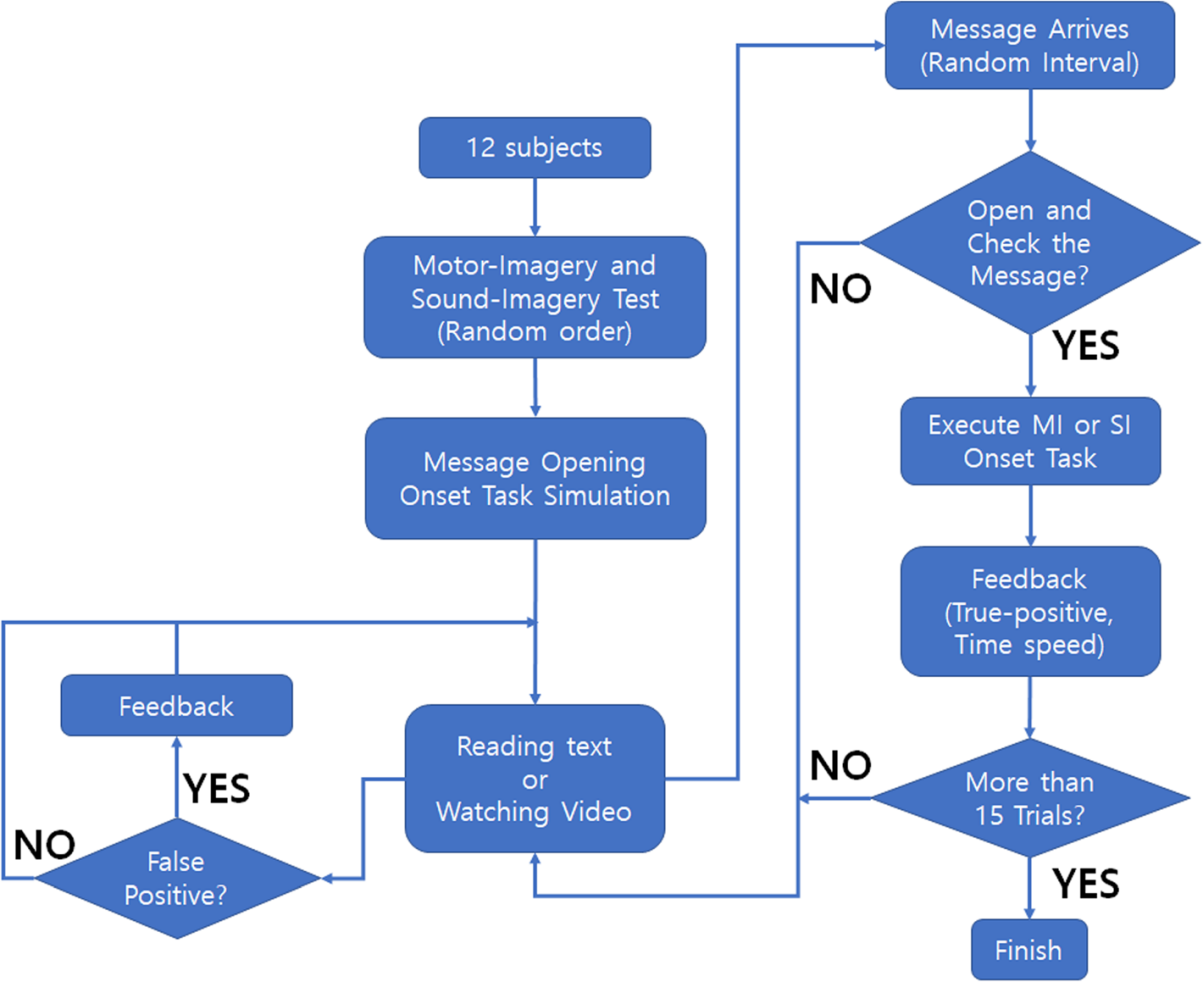
Block diagram of the experimental protocol

The experiment was designed to simulate a message opening system when a new message (prompt) arrived during realistic daily-life task situations (i.e., watching video, reading text from a book) and browsing photos. Fig. 1 shows an example of the system interface. On the background of the screen, a video clip was playing, and a subject was watching it while panels (A), (B) and (C) were hidden from the screen. Once the new message arrived (randomly between 5 s and 15 s in order to prevent subject anticipation), panel (A) smoothly and slowly slid into the side of the screen in order to minimise any Visual Event-related Potentials (VEPs). Then, the participants could either keep watching the video without trying to open the message, or they could open the message dialogue (B) by executing the Sound Imagery (SI) task state. The participants could perform the SI task action at any point, as the system was self-paced. However, they were asked to execute their onset at least 2 s after the new message dialogue had appeared, in order to eliminate any other event-related potentials (e.g. negative potentials or P300) so that the results were entirely based on the SI tasks. While participants were executing their SI task, they could estimate how long it took them to open the message dialogue by referring to the time keeping interface (D). This circular progress bar continuously turned from a light grey to a dark grey colour for 12 s, followed by dark to light grey again. There were small marks at each 1 s interval so participants could estimate their task execution time. As a result, the users could provide feedback to the PC on whether its response was correct (True-Positive, TP) or not (False-Positive, FP) as well as the execution time if it was a TP. After this feedback, panels (A), (B) and (C) disappeared. The process from (A) to (C) comprised a trial and each single run consisted of 15 trials. Each participant had to go through three different runs (which featured different background daily-life tasks). Background daily-life tasks were randomly ordered for testing for each participant in order to prevent any sequence-dependent results. The block diagram in Fig. 2 summarises the experimental protocol.

### Daily-life task scenarios

There were three different experimental scenarios. In the first two scenarios, the participants were instructed to open a message dialogue (as explained above) while they were on two different daily life scenarios (one was watching video and the other was reading text). The above message opening onset detection system was tested separately on each of the two daily tasks. The last experimental scenario was the sliding image task. The participants were presented with an image and if they wanted to slide the image to see the next one, they executed the mental task. In this scenario, there was no external stimuli such as a message alert. Consequently, the participants controlled the system in a 100% self-paced approach. These three different experimental scenarios were chosen because they are very common scenarios for most people in real-life situations.

In terms of material, a documentary titled “BBC - The Blue Planet” [19] was used for the video watching task as it requires low cognitive load and emotional neutrality [20, 21]. For the reading task, a book titled “English Fairy Tales” [22] was used as it does not have any complex text and is emotionally neutral as well. Hence, the material had reduced cognitive loads for both native and non-native English speakers. In the sliding image task, natural images (wild background scenery without animals) from [23] were used for emotional neutrality.

### Signal pre-Processing & Artefacts Handling

An Enobio (dry electrode equipment [24]) system was used for data acquisition. Seventeen electrodes were placed on the head based on a 10–20 layout and 1 reference channel was recorded on the right-side earlobe. Three extra external channels were placed on the forehead and both the right and left temples (anterior-most edge of the temporalis muscle) based on [25] in order to detect an Electrooculogram (EOG) and Electromyogram (EMG) for artefact removal purposes. The sample rate was 500 S/s (equipment bandwidth: 0–125 Hz) in order to ensure that all the EEG rhythms, up to some high gamma band, could be analysed. High gamma waves have not been widely used in BCIs due to concerns over contamination with EMG artefacts. However, studies have shown high gamma activity is associated with language tasks [26–28]. It was therefore included in the experiments and EMG artefacts handling methods were applied to avoid EMG-related classification results.

EEG data were wirelessly transferred from the Enobio to a PC via Bluetooth. These EEG data were bandpass filtered (Butterworth filter, order 5) with cut-off frequencies at 4 Hz and 100 Hz followed by a notch filter (Butterworth filter, order 5) at 49–51 Hz in order to remove mains interference. Then, the data were segmented with a 0.5 s window length.

The segmented data underwent automatic EOG detection based on [29]. A Discrete Wavelet Transform (DWT) with a Haar mother wavelet (decomposition level 6) was applied to the external channel that was placed on the forehead. If the external channel’s data were detected as EOG artefacts, the data segment was rejected from further analysis. If there was no EOG artefact, the EEG data were passed on to the EMG artefact removal process.

For automatic EMG removal, the Blind Source Separation by Canonical Correlation Analysis (BSS-CCA) was used, which is a very common and widely used EMG removal technique in BCIs. BSS-CCA assumes mutually uncorrelated sources that are maximally auto-correlated. It can therefore be used to separate the brain signal from the muscle activity (mainly facial, body movements) sources as the muscle artefacts have relatively low autocorrelation compared to the brain signal (please see [30, 31] for a more detailed discussion of BSS-CA). The threshold of the autocorrelation coefficient ρ was chosen at 0.35 based on [32]. Then, these pre-processed and EOG/EMG artefacts-handled EEG data was used for feature extraction and classification.

### Feature Extraction & Classification

**Fig. 3:**
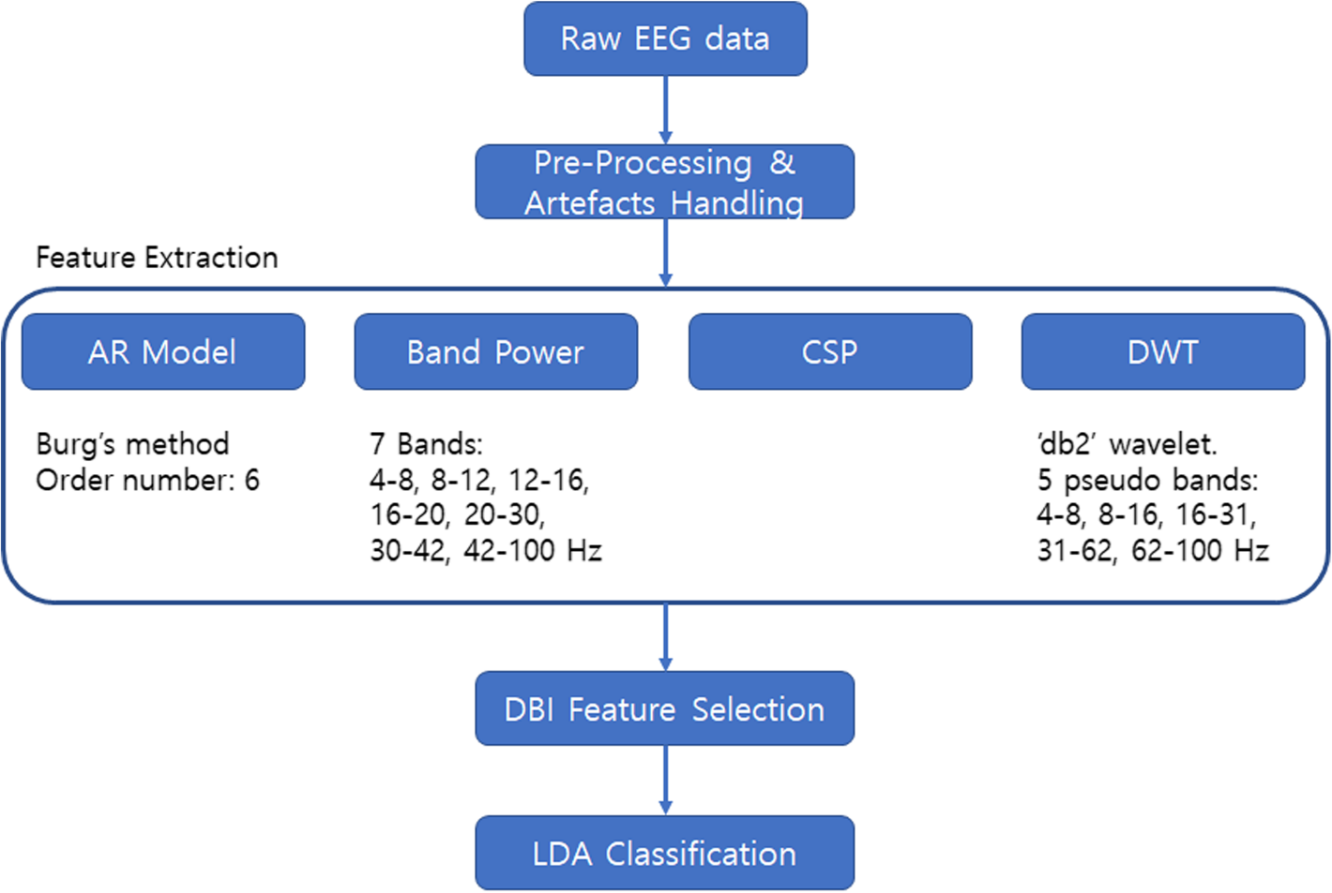
Feature extraction pipeline

Fig. 3 illustrates the feature extraction pipeline. In this experiment, four different feature extraction techniques were applied to the artefact-handled data. An ***AutoRegressive Model (AR model, Burg’s method)*** with order number 6 was applied based on [9]. The model coefficients were used as features. The second method was ***Band Power (BP)*** extraction with a Fast Fourier Transform (FFT). There were seven different bands: 4–8, 8–12, 12–16, 16–20, 20–30, 30–42 and 42–100 Hz. Each band’s FFT value was square powered and it was used as a feature. The third method was the ***Common Spatial Pattern (CSP)***. EEG source components were sorted in order to maximise the variance in one class and minimise it in the other class. Then, the first three and last three EEG source component variances were taken and linear regression was applied. The slope of the fitted line was used as a feature. The last feature extraction method was the ***Discrete Wavelet Transform (DWT)***. The data were decomposed up to level 7 and detailed parts, which represent the pseudo frequency bands of around 4–8, 8–16, 16–31, 31–62 and 62–100 Hz, were taken. From each detail part, the variance was calculated from the coefficients for dimensionality reduction. The mother wavelet ‘db2’ was chosen because of its common use in BCIs. These four different feature extraction techniques were chosen, as together they cover the time, frequency, spatial and time-frequency domains.

These feature extraction processes generated hundreds of feature points for each channel. Therefore, a feature selection method was required. In this experiment, the Davis-Bouldin Index (DBI [33]) was used. The optimal DBI threshold was calculated from the training data (training and validation set) for each subject and task. Firstly, we used the training data for optimal DBI threshold selection. Secondly, we divided the training dataset into training_2 and the validation set. Thirdly, we calculated the optimal DBI threshold number from 1 (increasing by 1). The validation set results show a gradual increase followed by a decrease. The peak point DBI value was chosen as the DBI threshold point for the testing data. Then, features that had DBIs below the threshold were used for classification.

For the classification, the Linear Discriminant Analysis (LDA) was used. It was chosen because of its simplicity and low computational cost [34]. Therefore, it suits the online classification for real-time processing as well as being a widely-used technique in BCIs.

### Performance evaluation method

**Fig. 4:**
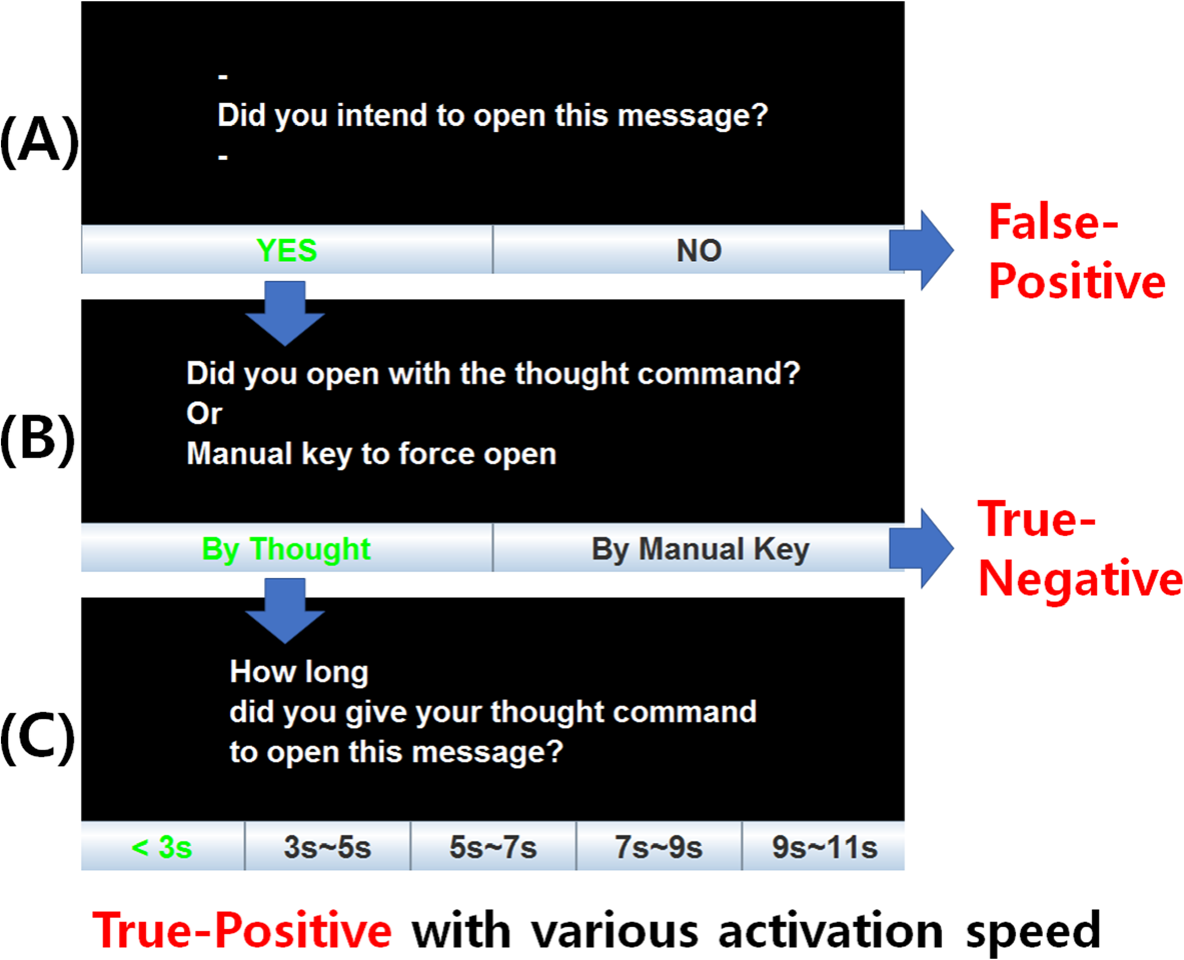
User feedback process during the online experiment for performance evaluation [8]

As the study was about an online onset detection system, a performance evaluation took place with the subjects’ feedback. Fig. 4 shows the feedback process. If the machine classified the incoming EEG data as an onset (intentional control) event from the user, a message window would appear on the screen with a feedback panel (A). The panel (A) could also be opened manually by pressing the ‘Esc’ button on the keyboard to indicate a True-Negative (TN) action. If the event was indeed an intended action, the user would choose ‘YES’. Otherwise the user would choose ‘NO’ for a False-Positive (FP). If the user chose ‘YES’, the feedback panel would change to (B) in order to clarify whether it was an actual thought command (i.e. sound-production) or a manual opening of the message (TN action). Subjects were asked to press the ‘Esc’ button when the continuous onset command (up to 11 s) did not work. If it was an intentional thought command, users were directed to panel (C) and were asked how much time had lapsed from the start of the onset until the message dialogue was opened. This will be regarded as a True-Positive (TP) with additional system response speed information (less than 3 s, 3–5 s, 5–7 s, 7–9 s or 9–11 s).

Based on the number of TP and FP, the ***True-Positive rate*** and ***False-Positive rate*** was calculated:

$$ {\boldsymbol{TP}}_{\boldsymbol{rate}}=\frac{\boldsymbol{number}\ \boldsymbol{of}\ \boldsymbol{TP}}{\boldsymbol{number}\ \boldsymbol{of}\ \left(\boldsymbol{TP}+\boldsymbol{TN}\right)}\kern1.25em ,\kern1.25em {\boldsymbol{FP}}_{\boldsymbol{rate}}=\frac{\boldsymbol{number}\ \boldsymbol{of}\ \boldsymbol{FP}}{\boldsymbol{Idle}\ \boldsymbol{event}} $$

The definition of the number of idle events is important for the calculation of the FP rate. Firstly, the idle period was defined as:

$$ \boldsymbol{idle}\ \boldsymbol{period}=\boldsymbol{total}\ \boldsymbol{recording}\ \boldsymbol{time}-\boldsymbol{task}\ \boldsymbol{activation}\ \boldsymbol{period}-\boldsymbol{refractory}\ \boldsymbol{period} $$

The refractory period is the period during which a signal is ignored for classification after the TP or FP action (i.e. while the message is opened for user feedback). Therefore, the total number of idle events which can yield output from the classifier was ***idle period (sec) / windows length (sec)***. In our case this was ***idle period / 0.5 s***. In addition, the ***True-False-Positive score (TFP***_***Score***_***)*** [35] was also calculated in order to take the idle period length into account for the final score in the self-paced system.

## Results

### Feature interpretation - spatial and spectral analysis for sound-imagery task

In this section, spatial and spectral characteristics will be analysed for the sound imagery task. As the experiment was online, this analysis was carried out with the training dataset, which was recorded as an offline setting.

**Fig. 5:**
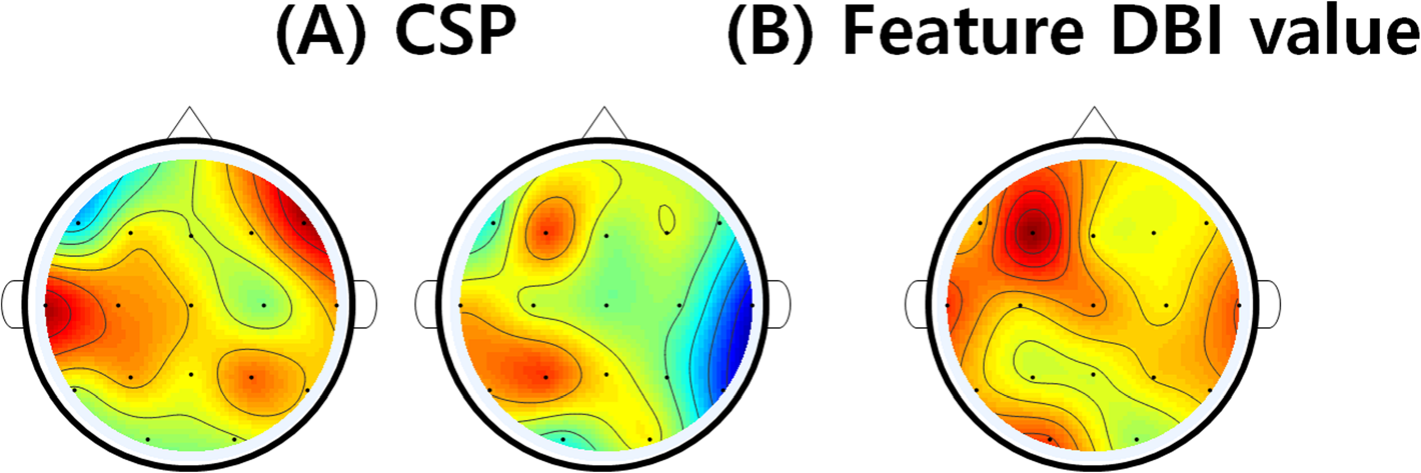
**a** Common spatial pattern averaged result. (Left: minimum variance for the idle period state. Right: minimum variance for the sound imagery task). **b** Spatial analysis with the DBI feature selection method

For the spatial analysis, the Common Spatial Pattern (CSP) was found based on the Enobio 17 channels electrode placement [24]. Fig. 5 (A) shows the visual pattern for the average result. The pattern varies depending on the subject because of the characteristic of EEG. However, the average result shows that channels around F3, P3 and T7, which are located near Broca’s and Wernicke’s area that are related to speech, had a big pattern difference between the idle and sound imagery task period. Similarly, channel F8 also showed some pattern difference.

In addition to the CSP analysis, the channels that had the best class separability based on the feature selection method are shown in Fig. 5 (B). From each participant, the 10 best feature points were selected based on the DBI feature selection method and their channel numbers were counted and summed up from twelve subjects. As can be seen from the Figure, channel F3 was selected the most amount of times as the best class separable channel, followed by channel T7. It shares some common results with the CSP spatial analysis by having the same F3 and T7 channels that are located near Broca’s and Wernicke’s area.

**Fig. 6:**
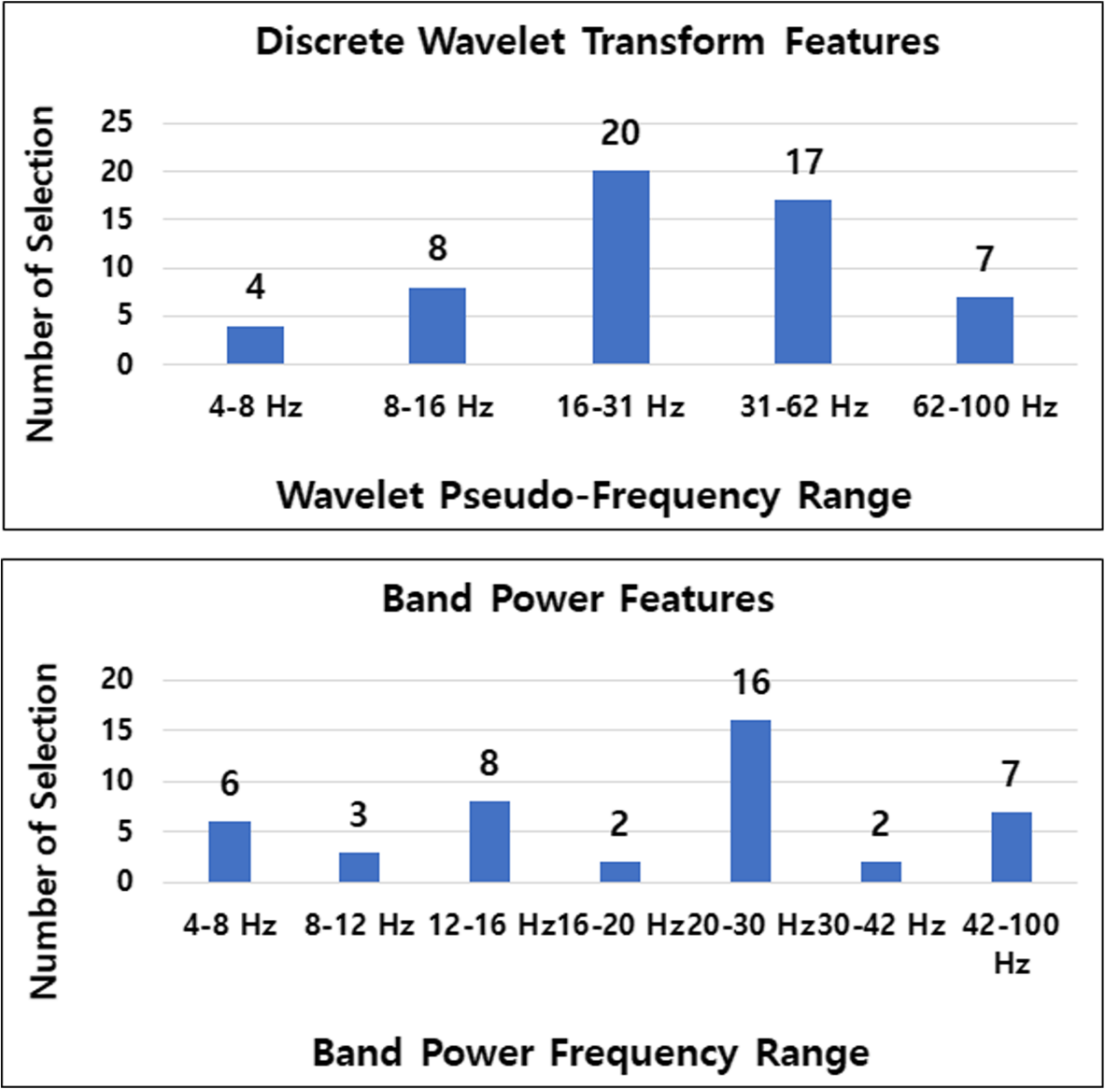
Spectral analysis with DBI feature selection method

In terms of spectral domain analysis, the frequency band that had the most class separability was found in a similar fashion. From the 120 feature points (the best 10 features from each of the twelve subjects), the wavelet transform feature was selected the most times (56 times), followed by the band power feature (44 times) and autoregressive model feature (20 times). The common spatial pattern feature was not selected at all be any of the subjects. From those 56 DWT and 44 band power features, frequency bands were counted to find out which range was selected the most as the best class separable frequency band. As can be observed from Fig. 6 (top part), the pseudo-frequency band of 16–31 Hz was selected the most times followed by the range of 31–62 Hz for the DWT feature. On the other hand, in the band power feature in the bottom part, the 20–30 Hz band was selected the most times. A review paper [36] reported that some studies suggested that the 30 Hz range should be elicited by linguistic processing of meaningful words but not of meaningless non-words. However, our high pitch sound imagery task showed the best class separability versus the idle state with the range of around 20–30 Hz.

### Sound-imagery vs. Motor-imagery for Onset Detection

Table 1 shows the classification performance with the True-Positive (TP) rate and False-Positive (FP) rate on both the Sound Imagery (SI) and Motor Imagery (MI) tasks in three different scenarios. In the sliding image task scenario, the twelve subjects’ average TP rate for the sound imagery task was 88.3% while the motor imagery task had a 73.3% rate. Only one out of twelve participants (P3) showed that the motor imagery task’s TP rate was higher than the sound imagery task’s and P5 showed the same TP rate with a lower FP rate in the sound imagery task. The Wilcoxon method was used for the statistical tests in this paper. It was chosen as it is a suitable test for our (non-parametric) data and it is widely used in BCIs. In terms of the Wilcoxon test *p* value, the sound imagery onset detection task had a significantly higher (*p* value at 0.033) TP rate than the motor imagery task. Even though the average FP rate in sound imagery had a lower value of 2.6% than the motor imagery at 4.8%, there was no statistically significant difference with a *p* value of 0.451.

**Table 1:** Online onset detection performance results in three different scenarios

	Sliding Image Scenario				Watching Video Scenario				Reading Text Scenario			
	Sound Imagery		Motor Imagery		Sound Imagery		Motor Imagery		Sound Imagery		Motor Imagery	
	TP rate (%)	FP rate (%)	TP rate (%)	FP rate (%)	TP rate (%)	FP rate (%)	TP rate (%)	FP rate (%)	TP rate (%)	FP rate (%)	TP rate (%)	FP rate (%)
P1	66.7	5.9	60	23.0	80	7.6	46.7	1.4	53.3	8.2	80	11
P2	100	4	93.3	3.2	93.3	1.5	73.3	2.6	100	1.7	73.3	2.5
P3	73.3	1	80.0	2.9	46.7	1.7	60	1.2	33.3	2.1	80	2.6
P4	93.3	9.4	86.7	3.9	100	6.9	86.7	4.3	93.3	4.2	86.7	6.7
P5	86.7	0.5	86.7	2.7	86.7	1.9	46.7	1.5	66.7	3.7	80	3.7
P6	80	5.5	60.0	5.2	86.7	6.8	60	1.5	73.3	5.5	73.3	2.6
P7	86.7	4	46.7	0.8	86.7	6.4	20	0.5	86.7	3.9	46.7	0
P8	93.3	0	33.3	0.0	73.3	0.7	20	0	100	2.6	66.7	0.1
P9	100	0.9	93.3	9.1	100	0.9	93.3	4.3	93.3	0.6	80	2.7
P10	93.3	0	73.3	0.5	86.7	0.6	73.3	0.2	86.7	0	86.7	0.5
P11	100	0	86.7	2.5	100	0	100	4.1	100	0.4	100	2.1
P12	86.7	0	80.0	3.5	93.3	6.5	86.7	1.5	86.7	2.5	73.3	1
Avg	88.3	2.6	73.3	4.8	86.1	3.40	63.9	1.90	81.1	2.90	77.2	3.00

In the video-watching scenario, the 12 subjects’ average showed an 86.1% TP rate for the SI task and 63.9% for the MI task. All the subjects had a higher TP rate with the SI than the MI task except for participant 3. The Wilcoxon test *p* value was 0.031, which depicts that SI had a significantly higher TP rate than the MI task. On the other hand, the average result of the FP rate shows that the SI task’s FP rate is slightly higher than the one of the MI tasks but there is no significant difference with a *p* value of 0.259. In the reading text scenario, the average TP rate of the SI task was 81.1 and 77.2% for the MI task. Even though the SI task showed a slightly better TP rate result, there was no statistically significant difference between them with a *p* value of 0.243. The FP rate also showed that the SI task provided a slightly better result (lower FP rate) but the difference was minor. If the two different daily-task scenarios are averaged, the TP rate of the SI task is significantly higher (83.6%) than the one of MI (70.6%) with a *p* value of 0.0106. There is, however, no significant difference with the FP rate.

In order to take into account all the true-positives, false-positives and the idle period length at the same time, as they are very important aspects of performance evaluation in self-paced BCI systems, the ***True-False-Positive score (TFP***_***Score***_***)*** presented in [35] was calculated and discussed here. 83.3% (10 out of 12) of the participants showed that the sound imagery onset detection task performed better in the TFP score than the motor imagery task in both the sliding image and watching video scenario. 66.7% (8 out of 12) of the participants showed a higher TFP score with the sound imagery task for the reading text scenario. Participant 3, who had previous experience in BCIs, showed that the motor imagery task performed better than the sound imagery task in all the daily-life scenarios but other participants, such as P4, P10 and P12, who also had BCI experience, did not follow the same pattern. Only two out of nine subjects showed that the MI task had a higher TFP score. From the naïve subjects, 87.5% (21 out of 24 cases) of them showed a higher TFP score with the SI task.

**Fig. 7:**
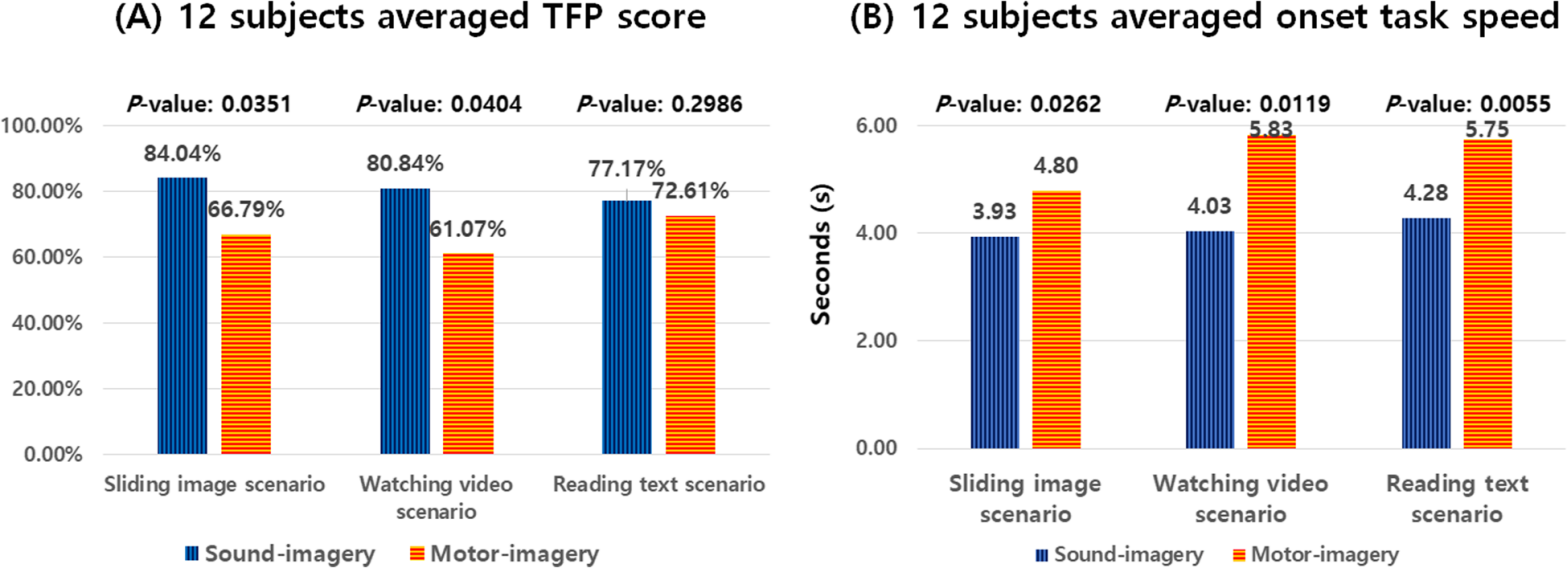
**a** Averaged True-False-Positive score result comparison between the SI and MI task in three different daily-life task scenarios. **b** Averaged onset system response speed comparison between the SI and MI

Figure 7 (A) shows the twelve subjects’ averaged TFP score for each daily-life task scenario. The sound imagery onset detection task produced a significantly higher TFP score than the motor imagery task with a *p* value of 0.035 and 0.04 for the sliding image and watching video scenario, respectively. However, there was no statistically significant difference for the reading text scenario even though the TFP score was higher for the SI task. The TFP score differences were very large in some cases. The image-sliding scenario had a more than a 17% difference (SI: 84.04%, MI: 66.76%) while the video watching scenario had a 19.77% difference (SI: 80.84%, MI: 61.07%). The text reading scenario, on the other hand, had only a 4.56% difference (SI: 77.17%, MI: 72.61%), and this difference was found to have no statistical significance (*p* = 0.298)

In terms of system response speed, the users’ feedback from Fig. 4 (C) was used in order to calculate the onset response time. Figure 7 (B) shows the twelve subjects’ averaged onset speed for the SI and MI tasks. The SI task required 3.93 s, 4.03 s and 4.28 s on average for the sliding image, watching video and reading text scenario, respectively, while the MI one required 4.8 s, 5.83 s and 5.75 s. In all of the three-different daily-life task scenarios, the SI task had a significantly faster onset response than the MI task by having a *p* value of 0.0262, 0.0119 and 0.0055, respectively.

## Discussion

This experiment investigated an online onset detection method for BCIs by prompting participants to open a message when it arrived in two different daily-life task scenarios (watching video and reading text) and in the sliding image task. Our new sound imagery task and typical MI task were tested and compared.

In terms of system performance, the sound imagery task achieved 84.04, 80.84 and 77.17% as a TFP score for the sliding image, watching video and reading text scenario, respectively, on average for twelve subjects. In contrast, the MI task achieved values of 66.79% (significantly worse), 61.07% (significantly worse) and 72.61% (no significant difference), respectively. In addition, the system speed showed a significantly faster response with the sound imagery than the MI task.

Although it is difficult to directly compare our results with other onset detection systems as the experiment environment and tasks are different, our SI task showed a relatively high TP rate. In [13], three subjects produced on average a classification TP accuracy of 79.67% between the motor-imagery task and the non-control state. In [37], six different mental tasks versus the idle state showed TP rates of between 55% (auditory imagery) and 72% (motor-imagery) on average over five subjects in an offline setting. Compared to these results, our 88.9% (in the video-watching case) and 78.9% (in the text-reading case) TP rates look very promising even though our study was carried out for more realistic scenarios than the ones previously reported by others.

From a usability point of view, participants completed a short survey at the end of the experiment regarding the level of difficulty of use of the two different SI and MI tasks. 0 depicts *very easy to use* and 10 represents *very difficult to use*. On average, SI received a value of 4.42 while MI received 6.42. Nine out of twelve (75%) subjects marked a lower value (easier to use) for the SI than the MI. Only participants P3, P4 and P12 said that the MI was easier. These three participants were BCI research students, who had experience in MI but not SI. On the other hand, one BCI research student and all the other naïve subjects marked the SI as easier to use. The *p* value of the twelve subjects was 0.0108. Therefore, the SI task was significantly easier to use than the MI one for the onset detection of BCIs.

Based on these results, our new sound imagery task outperformed the motor imagery task for the self-paced onset detection BCI system not only in performance but also in usability and system speed. Therefore, this onset detection system prototype showed some strong potential in terms of the real-life application of BCIs (compared to the typical motor imagery task) and it will move the BCI field a significant step forward once it is developed further by improving current EEG recording issues such as practicality and usability.

Contrary to the other two real-life activities, the text-reading scenario showed no significant TFP score difference between SI and MI. This may be because sound production imagery is harder while reading as they use same brain region (Broca’s area) based on our spatial pattern analysis and silent reading literature [38, 39]. With this in mind, the limitations of the proposed approach specifically in text-reading and related activities need to be further explored. Also, BCI researchers have discussed the so-called “BCI Illiteracy” problem, which is that about 15 to 30% of users are not able to use a MI BCI [40, 41]. As the SI task uses different parts of the brain, it may produce different results in regards to the BCI Illiteracy problem. This would be an interesting investigation.

## Conclusions

The scope of this study was to investigate how well our new sound imagery task works for a self-paced onset detection system in real-life scenarios by comparing it to a typical motor imagery task. From a performance point of view, our novel sound imagery task showed a significantly better TFP score in the sliding image (84.04%) and watching video (80.04%) scenario (opening message onset task) than in the motor imagery task (66.79 and 61.07%, respectively). Furthermore, the reading text scenario also reported a higher performance result with our approach (77.17% SI vs 72.61% MI). Moreover, the sound imagery task showed a significantly faster system response (4.08 s SI vs 5.46 s MI on average for the three scenarios) and had a significantly better usability (easier to use) score than the motor imagery.

Based on these results, our novel sound imagery onset detection system outperformed the motor imagery one and it showed great potential. This could be a significant step forward for the BCI field which is mainly restricted to research-oriented indoor laboratory settings with the use of motor imagery and cue-based studies.

## Data Availability

The datasets generated and/or analysed during the current study are not publicly available due [privacy policy – individual bio (EEG) information] but are available from the corresponding author on reasonable request.
